# Sex Differences in Vitamin Metabolism and Their Role in Oxidative Stress Regulation and Cardiometabolic Health

**DOI:** 10.3390/nu17162697

**Published:** 2025-08-20

**Authors:** Joanna Wróblewska, Anna Długosz, Marcin Wróblewski, Jarosław Nuszkiewicz, Weronika Wróblewska, Alina Woźniak

**Affiliations:** 1 Department of Medical Biology and Biochemistry, Faculty of Medicine, Ludwik Rydygier Collegium Medicum in Bydgoszcz, Nicolaus Copernicus University in Toruń, 24 Karłowicza St., 85-092 Bydgoszcz, Poland; joanna.wroblewska@cm.umk.pl (J.W.); jnuszkiewicz@cm.umk.pl (J.N.); al1103@cm.umk.pl (A.W.); 2 Department of Food Industry Technology and Engineering, Faculty of Chemical Technology and Engineering, Bydgoszcz University of Science and Technology, 3 Seminaryjna St., 85-326 Bydgoszcz, Poland; anna.dlugosz@pbs.edu.pl; 3 Students Research Club of Medical Biology and Biochemistry, Department of Medical Biology and Biochemistry, Faculty of Medicine, Ludwik Rydygier Collegium Medicum in Bydgoszcz, Nicolaus Copernicus University in Toruń, 87-100 Toruń, Poland; 316714@stud.umk.pl

**Keywords:** cardiometabolic health, metabolic syndrome, oxidative stress, sex differences, supplementation, vitamin

## Abstract

Vitamins A, D, E, K, B2, B12, and C play a key role in regulating metabolism and oxidative stress, significantly impacting cardiometabolic health. This review uniquely integrates mechanistic and epidemiological data to examine sex-specific differences in the bioavailability, metabolism, and physiological effects of these vitamins. By linking hormonal and genetic factors with oxidative stress modulation, lipid metabolism, and endothelial function, we outline how individualized vitamin intake strategies may help prevent cardiovascular and metabolic disorders. The paper also identifies natural dietary sources and optimal intake recommendations for each vitamin, emphasizing the importance of tailoring supplementation to sex-related needs. This sex-focused perspective provides a basis for developing personalized nutrition approaches to optimize cardiometabolic outcomes.

## 1. Introduction

Oxidative stress may lead to excessive production of reactive oxygen species (ROS), which damage proteins, lipids, and DNA, contributing to the development of various chronic diseases, including cardiovascular diseases, hypertension, and metabolic syndrome [1,2]. Cardiometabolic diseases, including hypertension, type 2 diabetes, and associated risk factors such as obesity and components of metabolic syndrome, are closely linked to oxidative stress. Hypertension represents one of the significant risk factors for cardiovascular diseases [3,4]. Oxidative stress is considered a key mechanism contributing to hypertension. Observational studies suggest that individuals with lower blood pressure may have higher levels of antioxidants such as vitamins A, D, E, and C, which could play a role in blood pressure regulation [5,6,7,8]. Considering sex differences in vitamin metabolism may allow for more effective strategies in the prevention and treatment of cardiometabolic diseases.

Vitamin metabolism is crucial in regulating oxidative stress and cardiometabolic health, with sex differences significantly influencing these processes. Women of reproductive age exhibit higher activity of antioxidant enzymes, such as superoxide dismutase (SOD), providing better protection against endothelial damage. In contrast, hypertension in men is more frequently associated with excessive NADPH oxidase (NOX) activity, leading to increased ROS production and impaired vascular function [5]. Use of antioxidant vitamin supplements is associated with a more favorable cardiometabolic health profile, including improved lipid levels, glucose control, and reduced inflammation, particularly among long-term multiple supplement users. [6]. Moreover, vitamin D is crucial in calcium–phosphate homeostasis, influencing vascular function and blood pressure regulation. Its deficiency can lead to elevated parathyroid hormone levels, promoting calcium deposition in vascular walls and increasing the risk of atherosclerosis [9]. Vitamin B12 levels are positively associated with better endothelial function, suggesting a potential role in maintaining vascular health in adults with type 1 diabetes at high cardiovascular risk [10]. The metabolism of vitamins A, B2, B12, C, D, E, and K plays a crucial role in regulating risk factors for cardiometabolic diseases [11,12,13,14,15,16,17]. Studies have shown that excessive adipose tissue accumulation disrupts the redox balance, potentially leading to endothelial damage, inflammation, and insulin resistance [18]. In experimental hypertension models, males have been shown to exhibit higher levels of oxidative stress compared to females, which may explain their stronger response to antioxidant therapy [5]. Sex-based differences may influence oxidative stress and antioxidant defense, with men showing greater cardiometabolic risk and women more often engaging in health behaviors such as vitamin supplementation, which is associated with improved inflammatory and lipid profiles [6,19].

Vitamins play a crucial role in maintaining cardiometabolic health, and both deficiencies and, in some cases, excess intake have been linked to various chronic diseases, including cardiovascular diseases and metabolic syndrome. Despite extensive research on their effects on the human body, many uncertainties remain regarding their mechanisms of action and sex-specific differences in the body’s response to vitamins. The synergistic action of specific vitamins is essential for reducing oxidative stress and supporting cardiovascular health. The combined supplementation of vitamins C and E has been shown to lower oxidative stress and inflammation markers [7]. However, excessive vitamin E intake may negatively affect the active form of vitamin K, disrupting its crucial role in blood coagulation regulation [4].

The requirements for vitamins A, D, E, K, B2, B12, and C differ between women and men due to distinct physiological, hormonal, and body composition factors. Men, having greater muscle mass, exhibit higher demands for vitamins in energy metabolism, whereas women, due to the menstrual cycle, pregnancy, and lactation, require increased amounts of vitamins that support hematopoietic functions and fetal development. Hormonal differences, such as the influence of estrogen on calcium absorption in women or testosterone on zinc requirements in men, also play a significant role in vitamin metabolism [20,21]. Additionally, the use of oral contraceptives can affect the levels of specific vitamins, necessitating their consideration in supplementation strategies [22,23].

This review article aims to demonstrate the relationship between sex differences in vitamin metabolism and their role in regulating oxidative stress and cardiometabolic health. In this context, it is essential to determine the recommended daily vitamin intake for women and men and identify their natural dietary sources, considering individual physiological needs, hormonal changes, and specific life stages. The manuscript focuses on these selected vitamins due to their well-documented effects on oxidative stress and cardiometabolic health in mechanistic and epidemiological studies. Only two B-group vitamins are described, as there are no consistent data regarding sex differences for the others. Proper food selection tailored to the body’s requirements is crucial for maintaining an optimal vitamin balance. A comprehensive understanding of the relationship between vitamin requirements and bioavailability enables dietary optimization, which, in turn, supports overall health, improves metabolic profiles, and prevents deficiencies that may lead to health disorders [24,25,26].

This review was conducted according to the principles of narrative and systematic literature review. A comprehensive search of the PubMed, Google Scholar, and Web of Science databases was performed up to July 2025. The search strategy combined free-text words related to vitamins (A, D, E, K, B2, B12, and C), oxidative stress, cardiometabolic health, and sex differences. Example search terms included “vitamin A” OR “retinol” OR “carotenoids”, “vitamin D” OR “cholecalciferol”, “vitamin E” OR “tocopherols”, “vitamin K” OR “menaquinones”, “riboflavin” OR “vitamin B2”, “cobalamin” OR “vitamin B12”, “ascorbic acid” OR “vitamin C”, combined with “oxidative stress”, “cardiometabolic health”, “cardiovascular disease”, “sex differences”, and “gender”. Inclusion criteria encompassed original studies (clinical trials, cohort studies, case–control studies, cross-sectional studies, and experimental models) and reviews that assessed sex-specific differences in vitamin metabolism, bioavailability, or clinical outcomes related to oxidative stress and cardiometabolic health. Only articles published in English or Polish were considered. Exclusion criteria included conference abstracts, case reports, editorials, and studies without sex-stratified analyses. References of relevant articles were hand-searched to identify additional publications.

## 2. The Impact of Vitamin A on Metabolic and Cardiovascular Health

Vitamin A and its metabolites play a crucial role in regulating various biological processes, including cell differentiation, cell cycle control, immune regulation, vision, and the maintenance of skin and mucosal health [27,28]. Moreover, they influence adipose tissue regulation by participating in adipogenesis and lipid and glucose metabolism, which may be significant in developing metabolic disorders such as obesity and insulin resistance [27,29]. The metabolism of vitamin A depends on transport proteins such as retinol-binding protein (RBP) and prealbumin (PA), which are responsible for its distribution in the body. RBP is the primary transporter of retinol, synthesized mainly in the liver. When bound to PA, it forms a stable retinol–RBP–PA complex, protecting retinol from renal filtration and extending its half-life in the serum [28,30]. Vitamin A is a fat-soluble compound in the body in several forms, including retinol, retinaldehyde, and retinoic acid [27]. In the body, two main isomers of retinoic acid are distinguished: all-trans-retinoic acid (atRA) and 13-cis-retinoic acid (13-cRA, isotretinoin) [2]. Among them, atRA is the most biologically active form, as it directly binds to retinoic acid receptors, regulates gene expression, influences adipogenesis, and modulates lipid metabolism [27,28]. Carotenoids, such as β-carotene, α-carotene, and β-cryptoxanthin, are the primary plant-derived precursors of vitamin A. Their bioavailability and conversion efficiency into retinol in the body depends on various factors, including their chemical structure and the presence of dietary fats. Studies have shown that β-cryptoxanthin and α-carotene may be more efficiently converted into retinol than β-carotene, suggesting their contribution to the body’s vitamin A supply may be underestimated [31]. In addition to serving as precursors to vitamin A, carotenoids have been observed to exhibit a protective effect against excessive adipose tissue accumulation, particularly in overweight individuals, and are associated with lower concentrations of inflammatory markers such as interleukin 6 (IL-6), interleukin 1 beta (IL-1β), and tumor necrosis factor-alpha (TNF-α). Additionally, the intake of these carotenoids is linked to increased activity of interleukin 10 (IL-10), which has anti-inflammatory properties. They also influence antioxidant mechanisms, increasing total antioxidant capacity and reflecting the body’s ability to neutralize oxidative stress [32].

Vitamin A neutralizes ROS, reducing cell oxidative damage and limiting lipid peroxidation [33]. Although vitamin A is not a direct antioxidant, it exhibits antioxidant properties by activating the Kelch-like ECH-associated protein 1/nuclear factor erythroid 2–related factor 2/antioxidant response element (Keap1/Nrf2/ARE) pathway, leading to the induction of phase II enzymes, which play a crucial role in neutralizing ROS and protecting against oxidative stress. Unlike conventional antioxidants, these enzymes are not rapidly depleted but act as catalysts, providing long-term cellular protection [7]. By supporting antioxidant and regulatory mechanisms, vitamin A may help reduce oxidative stress although its effects may vary depending on individual health conditions such as hypertension [34]. Consequently, an increasing number of studies suggest that vitamin A homeostasis plays a significant role in the development of these disorders, including metabolic syndrome (MetS), which increases the risk of cardiovascular diseases. In particular, RBP4, as the primary retinol-transporting protein, may serve as a biomarker of metabolic risk, as its elevated levels are correlated with obesity, insulin resistance, and an increased risk of cardiovascular diseases. Moreover, genetic and epidemiological studies suggest that the impact of vitamin A on MetS risk may vary depending on sex [28,35]. A study conducted by Ruiz-Castell et al. [36] demonstrated that higher vitamin A concentrations in women were associated with an increased risk of MetS. In contrast, no such relationship was observed in men. The authors suggest this may be due to sex-specific differences in vitamin A and lipid metabolism and varying adiponectin levels, a hormone with anti-inflammatory properties. Söderlund et al. [37] demonstrated that women have higher serum concentrations of all-trans retinoic acid, whereas men exhibit higher levels of 13-cis retinoic acid and retinol. These differences may be influenced by sex-specific regulatory mechanisms of retinoid metabolism, including the effects of sex hormones. Additionally, the higher retinol levels in women appear to be associated with a more stable antioxidant status, which may suggest greater susceptibility of men to oxidative stress and its consequences, including an increased risk of metabolic diseases [38]. Both vitamin deficiencies and excess intake can influence the risk of MetS. Studies have shown that low serum levels of carotenoids and vitamin C are associated with an increased risk of MetS. In women, excessive levels of vitamins A and E have been associated with a higher risk of MetS [39]. In men, suboptimal vitamin A intake is associated with higher body fat levels and an unfavorable lipid profile, which may be influenced by polymorphisms in the genes *Scavenger Receptor Class B Member 1* (*SCARB1*), *Uncoupling Protein 1* (*UCP1*), and *Uncoupling Protein 2* (*UCP2*). Carriers of specific alleles of these genes exhibit greater susceptibility to the adverse effects of insufficient vitamin A intake, manifesting as increased fat accumulation and altered expression of genes involved in lipid metabolism, oxidative stress, and thermogenesis [35]. Studies indicate that vitamin A levels and its transport proteins (RBP, PA) are associated with muscle mass and metabolism. Individuals with type 1 diabetes often exhibit reduced concentrations of these proteins, which may result from the influence of insulin on their synthesis and increased oxidative stress. Lower levels of creatinine and Triglycerides (TG) correlate with decreased PA, RBP, and retinol values, suggesting that muscle mass loss and lipid disturbances may affect vitamin A homeostasis. This may be particularly relevant in metabolic disorders such as diabetes and MetS [30].

Vitamin A can have both protective and harmful effects on the cardiovascular system, depending on its levels in the body. The study by Liu et al. [40] demonstrated that higher concentrations of atRA were inversely correlated with the risk of MetS. Individuals with lower atRA levels exhibited a worse cardiometabolic profile, including higher levels of inflammatory markers (C-reactive protein, (CRP), and IL-6) and oxidative stress (8-iso-prostaglandin F2α (8-iso-PGF2α)), as well as lower high-density lipoprotein cholesterol (HDL-C) concentrations. During a four-year follow-up, lower atRA levels were identified as an independent predictor of MetS development. Similar findings were reported in the China Health and Nutrition Survey (1997–2015), which showed that higher vitamin A intake was correlated with a lower risk of multiple cardiometabolic disorders. The protective effect was more substantial in women and individuals over 44, suggesting that vitamin A metabolism may be regulated by both hormonal factors and age-related metabolic changes [15]. Schiborn et al. [34] demonstrated that retinol and RBP4 levels influence the risk of type 2 diabetes and cardiometabolic diseases, depending on sex and participants’ health status. In women, elevated RBP4 levels were associated with a higher risk of type 2 diabetes, whereas no such relationship was observed in men. In contrast, Galmés et al. [41] found that low retinol intake correlated with an increased cardiometabolic risk in men. Individuals with the lowest intake had more than twice the risk of developing at least two metabolic risk factors compared to those with the highest vitamin A intake. Studies have shown that both β-carotene and retinol can influence metabolic health. β-carotene intake is beneficial for cardiometabolic health, whereas excessively low and high levels may increase the risk of these disorders [15]. Retinol exhibits a more substantial protective effect than β-carotene; however, once a certain level is reached, its beneficial impact does not increase further [15]. Notably, the protective effect of β-carotene is more pronounced in men, which may be due to their greater vascular susceptibility to oxidative damage and differences in antioxidant metabolism [7]. In contrast, higher intake of antioxidant vitamins, such as β-carotene, did not always translate into health benefits in women. This may be due to interactions between estrogen levels and oxidative-inflammatory mechanisms, suggesting that the protective effects of β-carotene may be hormonally regulated [7,42]. Therefore, the metabolism of vitamin A may be regulated by hormonal mechanisms, which could explain the differences in susceptibility to atherosclerosis and other cardiovascular diseases between sexes [43].

**Figure 1:**
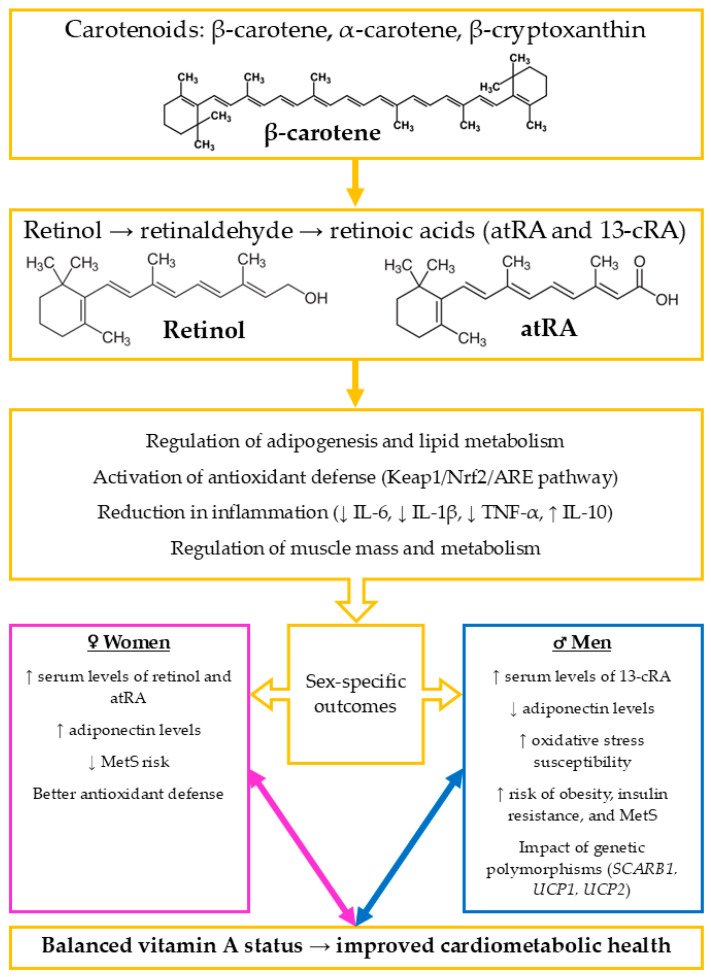
Schematic summary of vitamin A metabolism and its sex-specific effects on cardiometabolic health. Dietary provitamin A carotenoids-carotene, α-carotene, and β-cryptoxanthin—are converted into retinol, which is oxidized to retinaldehyde (retinal) as an intermediate and further metabolized to two main bioactive forms: all-trans-retinoic acid (atRA) and 13-cis-retinoic acid (13-cRA). These metabolites regulate key physiological pathways, including adipogenesis and lipid metabolism, antioxidant defense via the Kelch-like ECH-associated protein 1/nuclear factor erythroid 2-related factor 2/antioxidant response element (Keap1/Nrf2/ARE) signaling pathway, inflammation (by modulating interleukin-6 [IL-6], interleukin-1 beta [IL-1β], tumor necrosis factor-alpha [TNF-α], and interleukin-10 [IL-10]), and muscle metabolism. Sex-specific differences influence these effects; in women, higher serum levels of retinol and atRA, increased adiponectin levels, and stronger antioxidant capacity are associated with reduced risk of metabolic syndrome (MetS). In men, higher concentrations of 13-cRA, lower adiponectin, and greater susceptibility to oxidative stress contribute to increased risk of obesity, insulin resistance, and MetS. Additionally, genetic variants in *SCARB1* and *UCP1*, *UCP2* may exacerbate the metabolic impact of vitamin A imbalance. Maintaining adequate vitamin A status is essential for supporting cardiometabolic health, while both deficiency and excess can promote disease development. Increase (↑), decrease (↓), pink/blue boxes: sex-specific differences (men vs. women), orange: chemical forms and biological processes.

Figure 1 provides a schematic overview of the metabolic pathways, functional mechanisms, and sex-specific cardiometabolic effects of vitamin A, summarizing the key concepts discussed in this section.

## 3. Sex Differences in the Effects of Selected B Vitamins (B2 and B12) on Oxidative Stress and Cardiovascular Health

B vitamins are water-soluble organic compounds crucial in energy metabolism, DNA synthesis, nervous system function, and homocysteine regulation, essential for cardiovascular health [44,45].

Cobalamin (vitamin B12) is an example of a B vitamin that influences homocysteine levels by regulating its concentration, which is crucial for preventing cardiovascular diseases and maintaining DNA function [45,46]. A deficiency of this vitamin leads to elevated homocysteine levels, which may promote the development of atherosclerosis, hypertension, and other cardiometabolic diseases. Additionally, it negatively affects redox balance in the body, including the production of glutathione, which serves as an antioxidant [47,48,49]. Higher homocysteine concentrations in the body are strongly associated with an increased risk of developing cardiovascular diseases [49]. Low B12 levels have been associated with an unfavorable lipid profile, including elevated low-density lipoprotein cholesterol (LDL-C) and TG levels, which may increase the risk of cardiovascular diseases [46,50,51]. B12 supplementation has been reported to improve lipid profiles in some studies, potentially by reducing homocysteine, TG, and Total cholesterol (TC) levels while increasing HDL-C. Additionally, it exhibits anti-inflammatory effects [46,52]. According to the study by Vats et al. [42], vitamin B12 deficiency has been associated with an increased risk of ruptured abdominal aortic aneurysm, particularly among women with low intake levels. In contrast, Kayhana et al. [50] found that individuals with low serum vitamin B12 levels had higher systolic and diastolic blood pressure, elevated CRP and uric acid levels, and a higher prevalence of left ventricular hypertrophy than the control group. This suggests that serum vitamin B12 plays a crucial role in lipid metabolism and overall cardiovascular and inflammatory health, particularly in individuals with its deficiency.

Riboflavin (vitamin B2) plays a significant role in energy metabolism and influences the risk of cardiometabolic diseases, although its effects vary by sex. According to Wu et al. [46], higher vitamin B2 intake has been associated with a reduced risk of developing metabolic syndrome, particularly in men, through the regulation of blood pressure, glucose metabolism, and lipid profiles. In contrast, the study by Shin et al. [53] indicates that insufficient riboflavin intake, particularly in postmenopausal women, is associated with a higher risk of developing hypertension, diabetes, and metabolic syndrome. Riboflavin also supports antioxidant defense by activating enzymes such as SOD, reducing ROS levels, and helping protect DNA from oxidative damage. Additionally, riboflavin inhibits the activity of matrix metalloproteinase-9 (MMP-9), an enzyme that degrades the extracellular matrix and promotes the development of aortic aneurysms [54]. Vats et al. [42] demonstrated that higher vitamin B2 intake reduces the risk of developing abdominal aortic aneurysm (AAA) in men, particularly in carriers of the *NOX3* genotype variant, a gene encoding an enzyme responsible for ROS production. This suggests that the modulation of oxidative stress by riboflavin, through its effects on SOD, may contribute to lowering the risk of AAA development. Additionally, the anti-inflammatory effects of riboflavin include the reduction in TNF-α levels, which may be particularly significant in preventing hypertension and atherosclerosis, especially in women. Vitamin B2 supplementation represents a promising strategy for protecting the cardiovascular system and reducing oxidative stress in individuals at elevated risk [53,54].

Sex differences in B vitamin metabolism may result from varying dietary habits and hormonal influences. Differences in dietary habits, such as higher grain and meat intake among men and greater consumption of eggs and vegetables among women, may influence overall nutrient profiles and potentially affect riboflavin status and lipid metabolism. Additionally, hormonal changes, particularly the decline in estrogen levels in postmenopausal women, may exacerbate the adverse effects of B vitamin deficiencies, leading to an increased risk of hypertension, diabetes, and lipid disorders [53]. Both B vitamin supplementation in adulthood and their intake during childhood play a crucial role in the prevention of metabolic diseases, as B vitamin deficiencies may have long-term health consequences [51,55,56].

## 4. The Role of Vitamin C in Metabolic Health and the Cardiovascular System: Mechanisms of Action and Clinical Significance

Vitamin C, also known as ascorbic acid, is an essential nutrient involved in numerous biochemical processes in the human body. It exists in two forms: L-ascorbic acid and L-dehydroascorbic acid. L-dehydroascorbic acid is the oxidized form, which can be converted back into L-ascorbic acid within the body. L-ascorbic acid is a potent antioxidant that neutralizes ROS and protects cells from damage [57]. Vitamin C participates in the regulation of ROS levels by inhibiting enzymes such as NOX and nitric oxide (NO) synthase, further enhancing its protective effect on the circulatory system [7]. It also plays a key role in the regeneration of oxidized vitamin E, restoring its antioxidant properties. This is possible due to vitamin C’s ability to donate electrons, which allows vitamin E to regain its antioxidant function. In this way, vitamin C enhances the effectiveness of other antioxidants [7]. Since this vitamin is water-soluble and readily excreted by the kidneys, no upper toxicity limit has been established for it [58].

One of the primary mechanisms through which vitamin C influences cardiometabolic health is its ability to eliminate ROS [39]. Vitamin C regulates the conversion of proline to hydroxyproline, which supports collagen synthesis and promotes improved vascular elasticity and cardiovascular function [59]. Studies indicate that low serum vitamin C levels correlate with an increased risk of MetS, particularly in the context of high oxidative stress and chronic inflammation, which in turn elevates the risk of metabolic and cardiovascular diseases [39]. Epidemiological studies suggest an inverse relationship between vitamin C intake and the risk of developing MetS. Vitamin C deficiency in overweight and obese individuals disrupts lipid metabolism and exacerbates insulin resistance, increasing the risk of type 2 diabetes [60]. Individuals with MetS have been observed to have lower plasma vitamin C levels compared to healthy individuals, which may indicate its heightened consumption in anti-inflammatory and antioxidant reactions [61,62]. Both men and women who consume higher amounts of this vitamin are less likely to develop MetS, though this effect appears to be more pronounced in men [39,63]. High-dose vitamin C supplementation (1000 mg/day) has been reported to improve some metabolic parameters; however, it does not consistently lead to significant reductions in inflammatory markers such as CRP, IL-6, or TNF-α [61]. Dietary vitamin C intake is inversely correlated with the risk of coronary heart disease and stroke. However, these benefits are more pronounced at moderate doses, with higher intake not providing additional protective effects [64].

Vitamin C potentially impacts blood pressure, making it a significant factor in the context of MetS [65]. Its mechanism of action includes promoting sodium excretion and enhancing nitric oxide bioavailability, which together may contribute to blood pressure reduction [7]. A meta-analysis of randomized clinical trials demonstrated that vitamin C supplementation leads to a moderate decrease in both systolic and diastolic blood pressure, particularly in individuals with hypertension [66,67]. Additionally, an inverse relationship between vitamin C concentration and diastolic blood pressure has been observed, suggesting its potential role in hypertension regulation [62]. However, a study by Kim et al. [68] suggest that long-term vitamin C supplementation does not significantly affect blood pressure. This may be due to the fact that blood pressure regulation depends on multiple factors, and the antioxidant action of vitamin C alone may be insufficient to achieve a hypotensive effect. Interestingly, the blood pressure-lowering effect of vitamin C appears to be more pronounced in individuals with hypertension, who often include older adults or those with increased cardiovascular risk [66]. Beyond its impact on blood pressure, some studies suggest that vitamin C supplementation, especially when part of multivitamin use, may be associated with a reduced risk of coronary artery disease in women. A large prospective study among female nurses found that regular multivitamin supplementation containing vitamin C was linked to a lower incidence of cardiovascular events, including myocardial infarction and ischemic heart disease [69]. However, it is important to note that this effect is not uniform across all populations, and further research is needed to clarify its impact on cardiovascular outcomes in different demographic groups [66]. Some studies suggest that cardiovascular protection is more evident when vitamin C is consumed as part of a diet rich in natural antioxidants, whereas supplementation with synthetic forms may not yield the same benefits [64]. This may be due to interactions between vitamin C and other bioactive compounds in fruits and vegetables. Additionally, vitamin C may improve endothelial function by increasing NO bioavailability, essential for preventing hypertension and cardiovascular diseases [58]. In patients with heart failure, plasma vitamin C levels have been shown to support arterial dilation by enhancing NO release [57]. Vitamin C may enhance the bioactivation of nitrate to nitric oxide by limiting oxidative degradation of NO and restoring endothelial function [70].

## 5. The Impact of Vitamin D on Cardiometabolic Health and Oxidative Stress in the Context of Sex Differences

**Figure 2:**
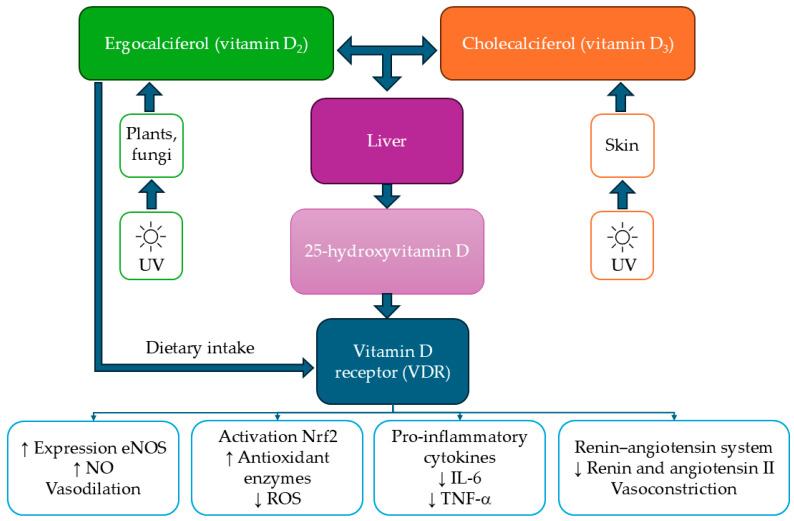
Mechanisms of action of vitamin D in terms of cardiometabolic health. Endothelial nitric oxide synthase (eNOS), Interleukin (IL), Nitric oxide (NO), Nuclear factor-erythroid 2 related factor 2 (Nrf2), Tumor necrosis factor-alpha (TNF-α), Ultraviolet radiation (UV), Increase (↑), Decrease (↓).

Vitamin D is a fat-soluble vitamin with two primary biological forms: ergocalciferol (vitamin D_2_) and cholecalciferol (vitamin D_3_). Vitamin D_3_ is synthesized endogenously in the skin upon exposure to UVB radiation (a specific range of ultraviolet light from the sun), whereas both forms can be obtained from dietary sources [71]. A key characteristic of vitamin D is its two-step hydroxylation process. First, the liver converts it into 25-hydroxyvitamin D (25(OH)D), the primary circulating form and biomarker of vitamin D status. Then, in the kidneys, it undergoes further hydroxylation to form the biologically active metabolite, 1,25-dihydroxycholecalciferol (calcitriol) [1,72]. This biologically active compound binds to the vitamin D receptor (VDR), regulating the expression of numerous genes and influencing various physiological processes [1]. The mechanisms through which vitamin D affects the cardiovascular system are multifaceted, including regulation of the renin–angiotensin system, anti-inflammatory effects, and modulation of endothelial function (Figure 2) [17]. Its deficiency is associated with endothelial dysfunction, an increased risk of atherosclerosis, and cardiovascular diseases [1]. One of the key mechanisms underlying this effect is the influence of calcitriol on NO bioavailability, which is crucial for activating guanylate cyclase in endothelial cells. This process leads to an increase in cyclic guanosine monophosphate levels in vascular smooth muscle cells. Elevated cGMP promotes intracellular sequestration of calcium ions (Ca^2+^) into storage compartments, thereby reducing their cytoplasmic concentration, weakening vascular smooth muscle contraction, and ultimately leading to vasodilation [1,73]. At the same time, vitamin D reduces ROS production, which can damage endothelial cells. Additionally, vitamin D inhibits NOX activity, whose excessive activation leads to increased ROS production and may exacerbate endothelial dysfunction [1].

Vitamin D promotes the activation of the nuclear factor erythroid 2–related factor 2 (Nrf2) transcription factor, leading to the upregulation of antioxidant enzymes such as SOD, glutathione peroxidase (GPx), and catalase, thereby enhancing the body’s antioxidant defense [2]. Additionally, vitamin D may regulate extracellular matrix metalloproteinases (MMPs), particularly MMP-2 and MMP-9, which play a crucial role in vascular remodeling and inflammatory processes [71]. By inhibiting the signaling pathways of the nuclear factor kappa-light-chain-enhancer of activated B cells (NF-κB) and reducing the production of pro-inflammatory cytokines such as TNF-α and IL-6, vitamin D exhibits an anti-inflammatory effect [1].

Interactions between vitamin D status and genetic predispositions may modulate the risk of vascular diseases, particularly in the context of sex differences [42]. Population studies have shown that men and women differ in serum 25(OH)D concentrations, which may influence their cardiometabolic health [74]. Sex hormones play a key role in modulating the expression of VDR and the enzymes responsible for its metabolism [1]. In women, anti-inflammatory mechanisms and interactions with estrogens may play a more significant role, as estrogens enhance VDR expression in the vascular system, potentially exerting a protective effect on vascular function [1]. Vitamin D deficiency reduces mitochondrial activity and decreases energy production, which may be particularly relevant for women during menopause when estrogen levels decline [75]. In contrast, in men, vitamin D deficiency is more frequently associated with an increased risk of hypertension and endothelial dysfunction, which may result from different hormonal regulation and lower VDR expression levels [1,42,75]. Women are more susceptible to vitamin D deficiency, which may also be attributed to their higher body fat percentage [74,76,77]. Increased adipose tissue promotes the sequestration of vitamin D, reducing its bioavailability and potentially leading to lower metabolic activity [78]. Vitamin D deficiency is a common phenomenon in the adult population and is associated with significant metabolic consequences, which may differ depending on sex [77,79]. Observational studies suggest a link between vitamin D deficiency, a serum 25OHD level below 50 nmol/L, and many prevalent diseases. However, it remains unclear whether these associations are causal. One of the key regulators of 25OHD levels is vitamin D-binding protein (VDBP), a group-specific component of serum globulin. VDBP is the primary carrier protein for 25OHD and the activated form of vitamin D, making it an essential reservoir for vitamin D metabolites. Approximately 85–90% of 25OHD is transported from the liver to target organs associated with VDBP. Higher 25OHD concentrations can lead to increased calcium absorption, which may subsequently affect its serum levels [80]. Sex differences may influence how vitamin D regulates glucose metabolism and insulin resistance. A high prevalence of vitamin D deficiency is observed in postmenopausal women, which may contribute to an increased risk of insulin resistance and metabolic disorders in this group [81]. Conversely, men may be more susceptible to the inverse relationship between low vitamin D levels and insulin resistance, potentially due to its role in modulating inflammatory processes [72]. Visceral adipose tissue, more commonly found in men, may further amplify this effect, exacerbating insulin resistance [76].

Vitamin D supplementation has been associated with beneficial changes in lipid profiles, particularly in women. Sharifi et al. [82] demonstrated that vitamin D supplementation was linked to reduced LDL-C and TC levels in women. In contrast, in men, TG increased, suggesting sex-specific differences in lipid metabolism. These differences may be related to sex-specific mechanisms, including hormonal effects and how vitamin D influences intestinal calcium and fat absorption, especially when calcium intake is low. When calcium intake is insufficient, vitamin D may enhance fat absorption, potentially increasing TC levels. Additionally, findings from Al-Daghri et al. [83] suggest that vitamin D may modulate apolipoprotein levels, potentially influencing lipid metabolism and sex-related differences in cardiometabolic risk. It was observed that after vitamin D supplementation, levels of apolipoproteins C1, C2, C3, and E increased, with apolipoprotein C1 rising significantly only in women, while apolipoproteins C2 and C3 increased only in men. Moreover, a reduction in apolipoprotein B levels was observed exclusively in women, which may explain the more favorable impact of vitamin D supplementation on lipid profiles in this group. Furthermore, the relationship between vitamin D status and cardiometabolic risk may be mediated by the amount and function of adipose tissue. Still, its action may depend on the amount and distribution of adipose tissue [84]. Vitamin D deficiency may also influence the regulation of VDR expression, disrupting adipocyte metabolism and promoting further fat accumulation [85]. Lower vitamin D levels are most commonly correlated with poorer metabolic indicators [72,76,79,84,86,87,88,89]. However, some reports suggest that higher vitamin D concentrations may be associated with elevated TG levels [88]. Additionally, cases have been described where individuals with higher vitamin D levels (despite still being classified as deficient) exhibit a better HDL-C profile [79]. Table 1 presents the relationships between vitamin D levels and cardiometabolic risk biomarkers, considering sex differences.

Vitamin D may support heart function by regulating calcium homeostasis and protecting mitochondria from oxidative stress [75]. The study by Sluyter et al. [16] demonstrated that vitamin D supplementation can benefit central blood pressure parameters, particularly in individuals with vitamin D deficiency, suggesting its potential role in cardiovascular protection. Vitamin D deficiency may impair heart function at the cellular level by reducing mitochondrial enzyme activity and weakening the ability of cells to adapt under metabolic stress conditions [75]. Long-term analyses have shown that individuals with the lowest vitamin D levels have a higher risk of overall and cardiovascular mortality, particularly an increased risk of death from coronary artery disease [74,90]. Specifically, the study by Kubiak et al. [91] indicates that, although vitamin D deficiency is associated with unfavorable health markers, supplementation in individuals with low 25(OH)D levels does not consistently lead to improvements in cardiometabolic outcomes. At the same time, observational evidence shows that individuals with low vitamin D concentrations are more likely to exhibit hypertension, experience myocardial infarction, and suffer other cardiovascular events [90,92]. The study by Sluyter et al. [16] indicates that these effects may be particularly pronounced in individuals with 25(OH)D levels below 50 nmol/L, where reductions in aortic pressure and improvements in vascular elasticity parameters were observed.

## 6. Sex Differences in the Effect of Vitamin E on Oxidative Stress and Cardiometabolic Health

Vitamin E is a group of organic compounds that includes tocopherols and tocotrienols. Structurally, it consists of a chromanol ring, responsible for its antioxidant properties, and a hydrophobic side chain that enables integration into lipid membranes and affects the bioavailability of different forms [4,93]. Tocotrienols, unlike tocopherols, have an unsaturated side chain, which grants them greater fluidity in biological membranes and potentially more potent antioxidant and anti-inflammatory properties [4]. As a powerful fat-soluble antioxidant, vitamin E plays a crucial role in protecting cells from oxidative stress [4,7,12]. Its mechanism of action involves neutralizing free radicals and safeguarding lipids, proteins, and DNA from oxidative damage [4,7].

Studies have shown that men exhibit higher oxidative stress levels than women, which may influence differences in response to vitamin E supplementation. In young, healthy men, oxidative stress markers such as thiobarbituric acid-reacting substances (TBARS) and 8-iso-PGF2α were found to be higher than in women, suggesting that men may have a greater need for antioxidants like vitamin E to neutralize ROS and prevent cellular damage [94]. Supplementation with antioxidant vitamins, including vitamin E, significantly reduced TBARS and 8-iso-PGF2α levels in men, whereas the effect was less pronounced in women [94]. Thus, differences in vitamin E levels may affect sex-specific responses to oxidative stress [12]. Vitamin E exerts protective effects on the endothelium by improving NO bioavailability, which facilitates vasodilation, and by reducing ROS levels, which can contribute to vascular dysfunction [7]. Studies indicate that vitamin E supplementation may lead to an average reduction of 3.4 mmHg in SBP [7], and that higher dietary vitamin E intake is associated with a significantly lower risk of hypertension in the general population [95]. However, the impact of vitamin E on cardiometabolic health remains inconclusive. A study conducted by Wannamethee [96] found that while plasma vitamin E concentrations were not associated with heart failure risk, higher dietary intake of vitamin E was linked to an increased risk of incident heart failure, particularly among older men without a prior history of myocardial infarction. Despite its antioxidant properties, vitamin E did not demonstrate a protective effect in this population, and in some cases, it may even pose a potential risk. Conversely, animal studies have shown that females have higher vitamin E levels in liver and heart tissues than males, suggesting that females may have better protection against oxidative stress in these organs. Epidemiological studies also indicate potential sex differences in the protective effects of vitamin E. Higher alpha-tocopherol concentrations have been linked to a lower risk of stroke in women but not in men [95]. A study conducted by Chae et al. [97] on healthy women with low cardiovascular risk found that long-term vitamin E supplementation was associated with a 40% reduction in the risk of heart failure with preserved ejection fraction. However, vitamin E did not significantly reduce the overall risk of heart failure or cardiovascular events such as myocardial infarction or stroke. High doses of vitamin E (over 400 IU per day) may increase overall mortality risk, raising concerns about its use in cardiovascular disease prevention [98].

## 7. The Role of Vitamin K in Cardiometabolic Health, Its Associations with Oxidative Stress, and Sex Differences

Vitamin K has two primary forms, K1 (phylloquinone) and K2 (menaquinones), which play a crucial role in blood coagulation, bone metabolism, and cardiovascular protection. Beyond its coagulation functions, it exhibits strong antioxidant properties by regulating oxidative stress levels through the modulation of enzymes such as GPx. Vitamin K2, particularly MK-7, can activate the Nrf2 pathway, enhancing protection against oxidative stress and inflammatory processes. Its deficiency is associated with an increased risk of cardiovascular diseases, highlighting its importance in maintaining redox homeostasis and metabolic health [99]. In the context of type 2 diabetes, vitamin K plays a significant role in glucose regulation and improving insulin sensitivity. Studies have shown that vitamin K1 and K2 supplementation can positively affect metabolic markers such as insulin levels, glycated hemoglobin, and HOMA-IR, indicating better glycemic control. Additionally, its effects may extend to lipid profile modulation, with some studies reporting reduced TG levels and favorable shifts in lipid fractions, which could help lower the risk of atherosclerosis and cardiovascular complications in patients with type 2 diabetes [13]. There are also significant sex differences in cardiovascular risk and response to anticoagulant therapy, which may indirectly relate to vitamin K dependent mechanisms. Women with cardiovascular diseases tend to exhibit higher levels of inflammatory markers and a stronger response to anticoagulant therapies. Studies on patients with atrial fibrillation indicate that women are more frequently classified as high-risk for thrombosis and stroke. This classification is based on clinical risk assessment systems considering age, hypertension, diabetes, cardiovascular diseases, and previous thromboembolic events. In these models, female sex is also regarded as an independent risk factor, making women more likely to qualify for anticoagulant therapy [100]. These findings underscore the need for an individualized approach to anticoagulant treatment and warrant further investigation into potential sex-related differences in vitamin K metabolism and its clinical implications.

## 8. Sex Differences in the Requirements, Bioavailability, and Absorption of Vitamins A, D, E, K, B2, B12, C, and Their Natural Sources in the Diet

The need for vitamins differs between women and men due to physiological, hormonal, and body composition differences. Men typically have more muscle mass and higher energy requirements, which results in a greater consumption of specific vitamins, particularly those involved in energy metabolism. On the other hand, due to the specificity of the menstrual cycle, pregnancy, and lactation, women have an increased need for iron, folic acid, and other vitamins crucial for hematopoietic function and fetal development [20]. Hormonal differences are key in vitamin metabolism and their bioavailability [22]. Estrogens in women improve calcium absorption, which may reduce the risk of osteoporosis. At the same time, testosterone in men increases the need for zinc, which is essential for synthesizing this hormone. Additionally, oral contraceptives can affect the levels of certain vitamins, such as B12, which should be taken into account when developing supplementation strategies [22,23]. Taking these relationships into account, the following analysis presents the recommended daily intake of vitamins for both women and men, as well as their natural sources in the diet [24,25,26]. The presented values of the Recommended Daily Allowance (RDA), Adequate Intake (AI), and Population Reference Intake (PRI) apply to healthy adults from 19 years of age, including those aged ≥ 75 years. They do not take into account infants, children, adolescents, pregnant women, or breastfeeding women.

According to guidelines from various institutions, the recommended daily intake of vitamin A is 700 µg for women and 900 µg for men in retinol or equivalent vitamin A units derived from beta-carotene. These standards align with the recommendations of the National Institute of Public Health-National Institute of Hygiene (NIZP-PZH) and the World Health Organization (WHO) [24,25]. The European Food Safety Authority (EFSA), which establishes the Population Reference Intake (PRI), sets values at 650 µg retinol for women and 750 µg for men, taking into account age and sex differences [26]. Natural sources of this vitamin mainly include animal products such as liver, eggs, and fatty fish (e.g., salmon, mackerel) and plant sources of beta-carotene, such as carrots, sweet potatoes, spinach, and kale. Vitamin A, being fat-soluble, requires the presence of fats in the diet for proper absorption. The absorption of beta-carotene (a precursor of vitamin A) from plant sources is lower than that of retinol from animal products. Vitamin A absorption can also be enhanced by other nutrients, such as vitamin C, which improves its absorption and conversion of beta-carotene to retinol [101].

The recommended daily intake of vitamin D for adults is 15 µg (600 IU), according to the guidelines of the NIZP-PZH and the EFSA, while the WHO recommends 10 µg (400 IU) [24,25,26]. Natural sources of vitamin D mainly include fatty fish (e.g., salmon, tuna, sardines), liver, egg yolks, and mushrooms, which can synthesize vitamin D after exposure to UV radiation. Natural vitamin D synthesis in the skin under sunlight also plays a crucial role, especially during summer. Vitamin D is fat-soluble, meaning its absorption is enhanced by fat in the meal. Vitamin D absorption from food can also be limited by gastrointestinal diseases, such as celiac disease or Crohn’s disease, which may interfere with fat absorption. In cases of insufficient sun exposure, supplementation with vitamin D is often necessary [102].

The recommended daily intake of vitamin E varies depending on the institution. NIZP-PZH sets the Recommended Daily Allowance (RDA) at 8 mg for women and 10 mg for men [24]. EFSA establishes the Adequate Intake (AI) at a higher level—11 mg for women and 13 mg for men [26]. WHO does not provide specific recommendations for vitamin E but suggests values similar to those recommended by NIZP-PZH, namely 8 mg for women and 10 mg for men [25]. Natural sources of vitamin E are primarily plant oils (e.g., wheat germ oil, sunflower oil), nuts (almonds, hazelnuts), and leafy green vegetables such as spinach and broccoli. Vitamin E is fat-soluble, and its absorption improves in the presence of fat in the diet. To increase its bioavailability, consuming vitamin E-rich foods with added fats (e.g., avocado or olive oil) is recommended. The absorption of vitamin E can also be limited by fat malabsorption disorders, such as cystic fibrosis or liver diseases [21].

The recommended daily intake of vitamin K differs depending on the institution. NIZP-PZH sets the AI at 55 µg for women and 65 µg for men [24]. EFSA recommends 70 µg for adults, while the WHO indicates higher values—90 µg for women and 120 µg for men [25,26]. Sources of vitamin K are mainly leafy green vegetables (e.g., kale, spinach, broccoli), olive oil, and fermented dairy products such as cheese. Vitamin K is fat-soluble, meaning its absorption also depends on the presence of fat in the diet. Fat malabsorption disorders like gastrointestinal diseases can limit vitamin K absorption. Additionally, supplementation with vitamin K in tablet form may affect its bioavailability significantly if supplementation is not correlated adequately with a diet rich in fats [103].

According to the guidelines of the NIZP-PZH, the recommended daily intake of vitamin B2 is 1.3 mg for adult men and 1.1 mg for adult women [24]. EFSA sets the daily requirement for riboflavin at 1.6 mg for men and 1.3 mg for women [26]. WHO recommends a daily intake of 1.3 mg for men and 1.1 mg for women, which aligns with the NIZP-PZH guidelines [25]. Significant sources of vitamin B2 include both animal and plant products. The richest sources of riboflavin are liver, dairy products, eggs, leafy green vegetables (e.g., spinach, broccoli), nuts, and whole grains. Riboflavin is absorbed in the upper part of the small intestine with the help of bile salts. Its metabolism and excretion are regulated by hormones such as triiodothyronine and aldosterone. After absorption, riboflavin is converted into active coenzyme forms flavin adenine dinucleotide and flavin mononucleotide, which are crucial in the body’s metabolic processes [104].

The recommended daily intake of vitamin B12 is 2.4 µg for both women and men, according to the guidelines of the NIZP-PZH, EFSA, and WHO [24,25,26]. Significant sources of vitamin B12 are animal products, such as meat, fish, eggs, and dairy. For vegetarians and vegans, supplementation with vitamin B12 may be necessary since this vitamin is found mainly in animal products. The absorption of vitamin B12 is complex and requires the presence of intrinsic factor (IF), which is produced in the stomach. Disorders in intrinsic factor production or gastrointestinal diseases like Crohn’s may lead to vitamin B12 deficiency. The absorption of B12 can also be hindered by interactions with other components, such as vitamin C, which may affect its bioavailability [105,106].

The recommended daily intake of vitamin C is 75 mg for women and 90 mg for men, according to the guidelines of the NIZP-PZH [24]. EFSA recommends higher values—95 mg for women and 110 mg for men, while the WHO provides a lower reference value of 45 mg daily for adults [25,26]. Sources of vitamin C are mainly fresh fruits and vegetables, especially citrus fruits, bell peppers, kiwi, strawberries, broccoli, and Brussels sprouts. Vitamin C is well absorbed in the gastrointestinal tract, especially in its natural form (e.g., from fruits and vegetables). Vitamin C absorption can be increased by consuming it in smaller, more frequent doses throughout the day, as the body has a limit for single-dose absorption. The intake of vitamin C with plant-based iron enhances the absorption of non-heme iron [107].

**Figure 3:**
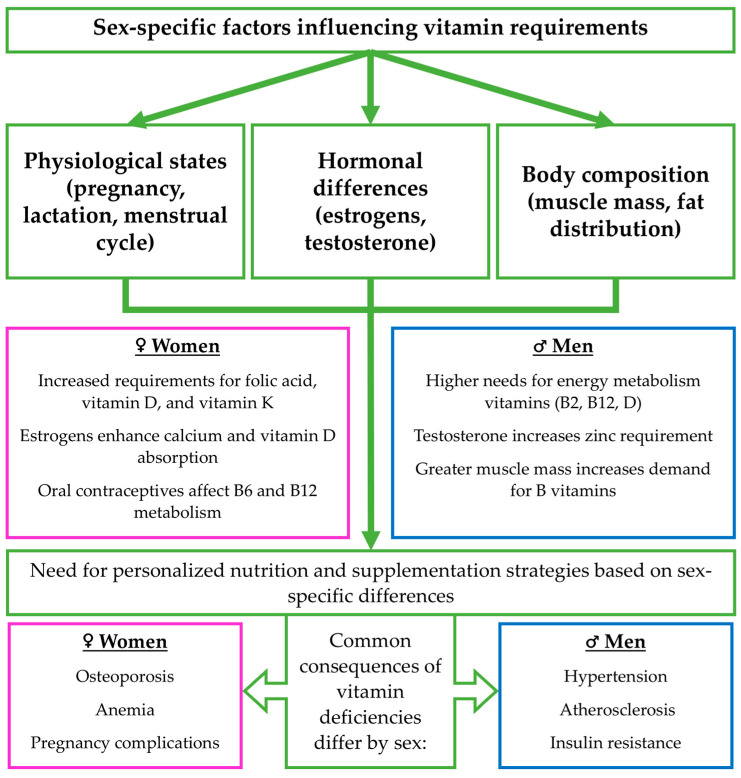
Summary of sex-specific factors influencing vitamin requirements, including hormonal differences (estrogens, testosterone), body composition, and physiological states (pregnancy, lactation, menstrual cycle). These factors contribute to differing needs for specific vitamins, such as folic acid, B6, B12, D, and K. The figure also presents key dietary sources of fat- and water-soluble vitamins and highlights how absorption may vary by sex. Differences in vitamin status can lead to distinct health outcomes, reinforcing the need for personalized nutrition and supplementation strategies. Pink/blue boxes: sex-specific differences (men vs. women), green boxes: factors and biological processes that influence vitamin requirements.

This analysis shows significant differences between women’s and men’s vitamin requirements, bioavailability, and metabolism. Hormonal, physiological, and body composition factors determine the specific nutritional needs of each gender. Women require special attention regarding vitamins essential for hematopoietic function and bone metabolism, while men need more vitamins that support energy metabolism. Deficiencies in key vitamins, such as D, E, and B12, may be linked to an increased risk of cardiometabolic diseases, including hypertension, atherosclerosis, and insulin resistance. Understanding these differences is key to optimal diet and supplementation planning, improving health, and preventing chronic diseases. A graphical summary of the key physiological, hormonal, and metabolic factors influencing sex-specific vitamin requirements, as well as their dietary sources and clinical implications, is presented in Figure 3.

## 9. Conclusions

The complex relationship between vitamin metabolism, oxidative stress regulation, and cardiometabolic health highlights the need to consider sex differences in research and clinical health approaches. The modulation of oxidative stress and inflammation by vitamins such as A, D, E, K, B2, B12, and C plays a key role in preventing cardiovascular and metabolic diseases. However, sex differences in these vitamins’ bioavailability, absorption, and metabolism significantly affect their effectiveness and impact on health outcomes. Women generally exhibit stronger antioxidant defense mechanisms, particularly during the reproductive years, while men are more susceptible to oxidative stress and related diseases, such as hypertension. Additionally, hormones and genetic factors contribute to the differing effects of vitamins, making a one-size-fits-all approach insufficient. Therefore, personalized strategies that consider these differences, especially in terms of intake and supplementation, could optimize prevention and treatment plans for cardiometabolic diseases. Recognizing sex-specific differences in vitamin metabolism provides an opportunity to design more effective, personalized nutritional and supplementation strategies. By aligning vitamin intake with hormonal status, body composition, and individual cardiometabolic risk profiles, clinicians and dietitians may improve the efficacy of preventive and therapeutic interventions. Such an approach can reduce the incidence of deficiencies or excesses, enhance antioxidant defense, and better address the unique needs of women and men across different life stages. Further research should focus on better understanding the mechanisms underlying sex differences and the adverse effects of both vitamin deficiencies and excesses, as well as on randomized clinical trials evaluating the impact of personalized vitamin supplementation, taking sex differences into account, in the context of mortality and cardiovascular events.

## Figures and Tables

**Table 1:** Relationships between vitamin D levels and cardiometabolic risk biomarkers considering sex differences.

Concentration	HOMA-IR	Insulin Level	TG	TC	LDL-C	HDL-C	Fasting Glucose Level	BMI	SBP	DBP	Ref.
Plasma 25(OH)D levels	IR ♂	IR ♂	IR ♂	IR♀	IR ♀	NA	NA	-	-	-	[72]
Serum 25(OH)D levels	IR ♀	NA	-	-	-	-	IR ♂	IR ♂ > ♀	NA	NA	[86]
Serum 25(OH)D levels	IR ♂ > ♀	IR ♂ > ♀	IR ♀ > ♂	IR ♀ > ♂	IR ♀ > ♂	NA	NA	IR ♂ > ♀	NA	IR ♂	[76]
Serum 25(OH)D levels	IR ♂	IR ♂	IR♂	IR♀	IR ♀ = ♂	PR ♀ = ♂	IR♀	IR ♀ = ♂	IR ♀ = ♂	IR ♀	[79]
Serum 25(OH)D levels	IR ♀ = ♂	-	PR ♀	NA	NA	NA	-	IR ♀ > ♂	NA	-	[88]
Plasma 25(OH)D levels	IR ♂	IR ♂	IR ♂ > ♀	-	NA	NA	NA	IR ♀ = ♂	NA	NA	[89]

Body mass index (BMI), Diastolic blood pressure (DBP), Effect more significant in women (♀ > ♂), Effect significant only in women (♀), Effect more significant in men (♂ > ♀), Effect significant only in men (♂), High-density lipoprotein cholesterol (HDL-C), Homeostatic model assessment insulin resistance (HOMA-IR), Inverse relationship (IR), Low-density lipoprotein cholesterol (LDL-C), No association (NA), No sex differences (♀ = ♂), Positive relationship (PR), Systolic blood pressure (SBP), Total cholesterol (TC), Triglyceride (TG).

## Data Availability

No new data were created or analyzed in this study. Data sharing is not applicable to this article.

## Abbreviations

The following abbreviations are used in this manuscript:

8-iso-PGF2α	8-iso-prostaglandin F2α
13-cRA	13-cis-retinoic acid
25(OH)D	25-hydroxyvitamin D
AAA	Abdominal aortic aneurysm
AI	Adequate Intake
atRA	all-trans-retinoic acid
CRP	C-reactive protein
DBP	Diastolic blood pressure
EFSA	European Food Safety Authority
eNOS	Endothelial nitric oxide synthase
GPx	Glutathione peroxidase
HOMA-IR	Homeostatic Model Assessment Insulin Resistance
IL-1β	Interleukin 1 beta
IL-6	Interleukin 6
IL-10	Interleukin 10
Keap1/Nrf2/ARE	Kelch-like ECH-associated protein 1/nuclear factor erythroid 2–related factor 2/antioxidant response element
LDL-C	Low-density lipoprotein cholesterol
MMPs	Matrix metalloproteinases
MetS	Metabolic syndrome
NIZP-PZH	National Institute of Public Health-National Institute of Hygiene
NOX	NADPH oxidase
NO	Nitric oxide
Nrf2	Nuclear factor-erythroid 2 related factor 2
PA	Prealbumin
PRI	Population Reference Intake
RDA	Recommended Daily Allowance
ROS	Reactive oxygen species
RBP	Retinol-binding protein
SBP	Systolic blood pressure
*SCARB1*	*Scavenger receptor class B member 1*
SOD	Superoxide dismutase
TBARS	Thiobarbituric acid-reacting substances
TC	Total cholesterol
TG	Triglyceride
TNF-α	Tumor necrosis factor-alpha
*UCP1*	*Uncoupling Protein 1*
*UCP2*	*Uncoupling Protein 2*
WHO	World Health Organization
VDBP	Vitamin D-binding protein
VDR	Vitamin D receptor
